# Isolation and characterization of *Lentilactobacillus diolivorans*: a high n-propanol-producing microorganism from Baijiu brewing

**DOI:** 10.3389/fmicb.2025.1624097

**Published:** 2025-06-25

**Authors:** Enhua Zhang, Ruijie Gao, Kai Zhu, Pulin Liu, Weifang Liao, Tuanyuan Yang, Xiaotong Liang, Menglu Yang, Yingying Ming, Lihong Miao

**Affiliations:** ^1^School of Life Science and Technology, Wuhan Polytechnic University, Wuhan, China; ^2^Hubei Baiyunbian Liquor Industry Co., Ltd., Songzi, China

**Keywords:** Baijiu, n-propanol, isolation, *Lentilactobacillus diolivorans*, quantitative PCR, fermentation characteristics, transcriptome analysis

## Abstract

It is now understood that n-propanol, a higher alcohol constituent in Baijiu, significantly impacts its overall quality. Excessive levels can impart a spicy and bitter taste, necessitating strict control. Despite its importance, the precise mechanism underlying the production of high-content n-propanol in Baijiu brewing and the specific microorganisms responsible for this process remain unclear. This study isolated *Lentilactobacillus diolivorans* ZX6, known for its high n-propanol production, from Baijiu fermented grains using a modified MRS (SMRS) enrichment medium and gas chromatography detection. The pure strain ZX6 produced 4,399 mg/L of n-propanol in SMRS medium. Using the key enzyme gene sequence for 1,2-propanediol metabolism in *L. diolivorans*, we designed primers and established a quantitative PCR method to quantify *L. diolivorans* in first-round fermented grains from three different sauce-flavor Baijiu distilleries. The widespread presence of *L. diolivorans* in sauce-flavor Baijiu fermented grains indicates its potential role as one of the key microorganisms responsible for the high concentrations of n-propanol. *L. diolivorans* ZX6 directly utilized glucose fermentation to produce large amounts of n-propanol. High n-propanol synthesis from *L. diolivorans* ZX6 required high sugar concentrations, anaerobic conditions, and extended fermentation times. Transcriptome analysis revealed that pyruvate and lactic acid, likely major precursors, could enter the methylglyoxal pathway under the catalysis of lactaldehyde dehydrogenase, respectively. These compounds were subsequently metabolized to 1,2-propanediol and then to n-propanol. These findings provide insights into identifying n-propanol-producing microorganisms and establish a theoretical basis for elucidating the high-yield n-propanol mechanism in Baijiu brewing, along with strategies for regulating its content.

## Graphical Abstract

Isolation, validation, and metabolic characterization of *Lentilactobacillus diolivorans* as a key producer of high n-propanol in Baijiu fermented grains. 
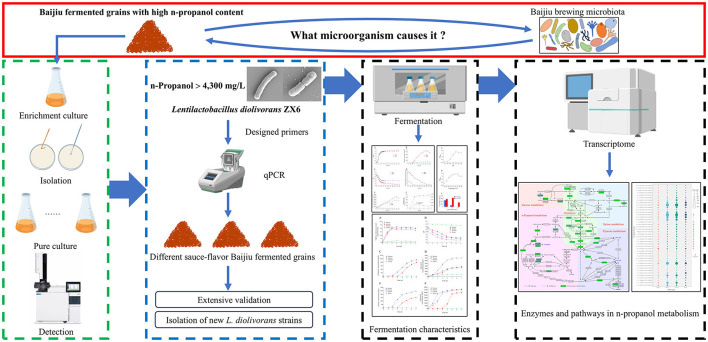

## 1 Introduction

Baijiu (Chinese liquor), one of the world's six renowned distilled spirits, derives its quality from its flavor components (Jin et al., 2017). Higher alcohols, mainly including n-propanol, isobutanol, and isoamylol, are key flavor compounds in Baijiu (Gao et al., 2014; Tian et al., 2020). Their concentration significantly impacts Baijiu's flavor profile and drinking comfort. While moderate levels enhance flavor, excessive amounts can lead to off-flavors and discomfort (Mckarns et al., 1997; Strubelt et al., 1999). Therefore, higher alcohol content is strictly regulated in Baijiu. Among higher alcohols, n-propanol can impart a spicy and bitter taste at high concentrations (Dong et al., 2002; Luo et al., 2025). Various microorganisms can synthesize n-propanol, with yeast being the primary contributor to higher alcohols (Giudici et al., 1990; Wu et al., 2016; Hu et al., 2021; Wei et al., 2024). Bacteria and molds also possess limited n-propanol production capabilities (Janssen, 2004; Siebert and Wendisch, 2015; Matsubara et al., 2016; Walther and François, 2016). Traditional Chinese Baijiu's multi-batch solid-state fermentation and distillation process involves a diverse microbial community, including bacteria, yeast, and molds. This complexity has made the identification of the specific microorganisms responsible for target flavor substances challenging (Liu et al., 2017; Hao et al., 2021; Tu et al., 2022; Wang et al., 2025).

Sauce-flavor Baijiu (SFBJ), a representative Baijiu type, is characterized by its stacking fermentation and pit fermentation, which involve multiple fermentation rounds and nine rounds of steaming (Zhou et al., 2023; Chen et al., 2024b). A common issue in SFBJ production is the abnormally high n-propanol content in the first and second distillation raw liquors, significantly affecting Baijiu quality. Baiyunbian Liquor, a widely consumed sauce-strong-flavor Baijiu, employs the production techniques of SFBJ in the first seven rounds. Its n-propanol content in the first and second raw liquors reportedly reaches 25,690 mg/L and 18,011 mg/L, respectively, constituting 92% of total higher alcohols (Dong et al., 2002). Gas chromatography analysis of SFBJ in the Maotai town, Guizhou province, revealed that the n-propanol content in the third raw liquor was as high as 3,247 mg/L, accounting for 94.1% of total higher alcohols (n-propanol, isobutanol, isoamylol) (Guo et al., 2022). The production of such high n-propanol levels in Baijiu fermented grains cannot be explained solely by the n-propanol yields of existing pure or mixed strains of microorganisms. Studies indicate that pure yeast strains produce < 80 mg/L (kg) of n-propanol during liquid and solid-state fermentation, with n-propanol accounting for < 50% of total higher alcohols (Giudici et al., 1990; Qu et al., 2021). Our mixed fermentation experiments with three yeast species (*Saccharomyces cerevisiae, Pichia kudriavzevii, Candida tropicalis*) and three lactic acid bacteria (LAB) species (*Lactiplantibacillus plantarum, Lentilactobacillus buchneri, Lactobacillus acetotolerans*) isolated from SFBJ fermented grains did not reveal significant synergistic effects on n-propanol production. There was also a phenomenon of abnormally high levels of n-propanol during Xiaoqu Baijiu (XQBJ) fermentation (Qu et al., 2021). Therefore, the microorganisms or microbial communities capable of producing high levels of n-propanol in the Baijiu fermentation system remain to be elucidated.

Although the content 006Ff n-propanol in the first round of fermented grains of SFBJ was higher than that of XQBJ, the sampling time was strictly limited by the production cycle of SFBJ. To identify and isolate potential high n-propanol-producing microbial strains within the Baijiu brewing system, we initially collected high n-propanol-containing XQBJ fermented grains and inoculated them into a liquid medium for enrichment culture, which was then followed by dilution plate separation. After isolation and characterization of single colonies, their n-propanol fermentation yields were determined to screen for high-producing strains. Using the target high n-propanol-producing bacterium's genome information, we designed specific PCR primers to target n-propanol synthesis genes. We employed fluorescent quantitative PCR (qPCR) to quantify the target bacterium in the first round of fermented grains from representative SFBJ, and then isolated and identified new target bacterium strains. Furthermore, we investigated the effects of culture medium sugar concentration and fermentation conditions on n-propanol production by the target strain, aiming to elucidate the external factors contributing to high n-propanol formation during Baijiu brewing. Finally, transcriptomic analysis of the target strain grown on different carbon sources was performed, revealing its potential metabolic pathways for n-propanol biosynthesis.

## 2 Materials and methods

### 2.1 Sample collection and sources

Fermented grain samples from the first round of SFBJ pit fermentation (30 days) were collected from three distilleries in three regions: Songzi, Hubei (D1, D2); Shennongjia, Hubei (SNJ-1, SNJ-2); and Maotai town, Guizhou (GZ-1, GZ-2) (Supplementary Table S1). XQBJ fermented grains (sample X) were collected from Songzi, Hubei. For each fermentation pit, 2 kg of fermented grains were collected from the upper, middle, and lower layers and then thoroughly mixed to form the composite sample for analysis. Samples were stored at −20°C or 4°C for subsequent flavor and microbial analysis. DNA was extracted from fermented grains with high n-propanol content, and *Lentilactobacillus diolivorans* quantification was performed. Stacking-fermented (48 h) samples and Aged Zaopei (distilled fermented grains from XQBJ pit fermentation) were obtained from Hubei Baiyunbian Liquor Industry Co., Ltd. (Songzi, China).

### 2.2 Microorganisms and media

*Limosilactobacillus panis* strain CGMCC 1.3925 was obtained from China General Microbiological Culture Collection Center. The LAB strains *L. diolivorans* ZX6, ZD9, ZD92, and ZD93 were isolated and identified from Baijiu fermented grains in this study. *S. cerevisiae* SCY62 was isolated from SFBJ fermented grains and stored in our laboratory. *Rhizopus* Qu (Angel *Rhizopus*), used as a saccharifying agent in XQBJ fermentation, was purchased from Angel Yeast Co., Ltd. (Yichang, Hubei, China).

MRS medium (Hopebio Company, Qingdao, China), containing 10 g/L peptone, 8 g/L beef extract, 4 g/L yeast extract, 20 g/L glucose, 5 g/L sodium acetate, 1 g/L Tween-80, 2 g/L dipotassium phosphate, 2 g/L ammonium citrate, 0.2 g/L magnesium sulfate, 0.04 g/L manganese sulfate, and adjusted to pH 6.5 ± 0.2, was used for culturing and enumerating the total number of LAB. Yeast extract-peptone-dextrose (YPD) medium, consisting of 20 g/L glucose, 20 g/L peptone, and 10 g/L yeast extract, was used for yeast culturing. Rose Bengal Agar (Hopebio Company, Qingdao, China) was employed for culturing and counting yeast populations.

Sorghum extract medium (SS) was used for fermentation and enrichment culture preparation. The sorghum extract preparation method was previously described in the literature (Deng et al., 2020). The obtained sorghum extract was diluted with water to achieve a final sugar concentration of 12 °Bx and adjusted to a pH of 5.8 using a 1:1 mixture of lactic acid to acetic acid. Fermentation media were sterilized at 121°C for 20 min. The modified MRS (SMRS) enrichment medium, a 1:1 mixture of SS and MRS medium with a pH of 6.1 ± 0.1, was used for n-propanol-producing microorganism enrichment and isolation. Nystatin and ampicillin trihydrate (Macklin Biochemical Technology Co., Ltd., China) were added at concentrations of 100 mg/L and 80 mg/L, respectively. Xiaoqu Baijiu solid fermentation medium (XBSM) consisted of 40% (w/w) fresh steamed sorghum (sorghum cooked and cooled with pH 6.95) and 60% (w/w) sterilized aged Zaopei (with pH 3.75). This medium was used for solid-state mixed fermentation of yeast and LAB.

### 2.3 Laboratory fermentation

To evaluate n-propanol production by *L. diolivorans* in liquid co-culture systems, *S. cerevisiae* SCY62 and *L. diolivorans* ZX6 were first cultured separately under the following conditions: SCY62 in YPD medium at 28°C with 180 rpm shaking for 24 h, and ZX6 in MRS medium under static conditions at 30°C for 72 h. Mixed-culture fermentations were conducted in 250 mL triangular flasks containing 150 mL of SMRS medium. The inoculation ratios (cells/cells) of *S. cerevisiae* SCY62 to *L. diolivorans* ZX6 were 1:0, 1:1, 1:0.1, and 1:0.01, respectively. The initial cell concentration of *S. cerevisiae* SCY62 in fermentation medium was 1 × 106 cells/mL. Static cultures were maintained at 30°C for 6 days.

Five hundred milliliters of plastic bottles were used as containers for solid-state fermentation. XQBJ solid-state fermentation was conducted using an XBSM medium. Twelve XBSM medium samples were inoculated with 5% (w/w) of *Rhizopus Qu*, 5 × 105 CFU/g of *S. cerevisiae* SCY62, and varying amounts of *L. diolivorans* ZX6 (0, 104, 105, and 106 CFU/g). The fermentation material was initially fermented by stacking in a plastic box at 28°C for 24 h, then transferred to 500 mL plastic bottles for anaerobic fermentation at 28°C for 10–20 days.

To simulate the pit fermentation of SFBJ, fermented grains that had completed stacking fermentation were collected from the Baijiu distillery, individually packed into 500 mL plastic bottles, and incubated. Five fermented grain samples from the first round of SFBJ stacking fermentation at Baiyunbian Liquor Industry Co., Ltd. were randomly selected and separated. Five groups labeled Pit No. 1 through No. 5 were established, each containing 15 parallel plastic bottles. Fifteen kilograms of mixed samples were taken from each stacking fermentation mash and distributed equally among the 500 mL plastic bottles in the five groups. The bottles were sealed and incubated at 28°C for 28 days. Gas chromatography and qPCR analyses were conducted on samples from each fermentation pit at 0, 7, 14, 21, and 28 days. Three sample bottles were taken at each time point for analysis. Additionally, these samples were used for LAB isolation (refer to Section 2.6).

### 2.4 Enrichment and isolation of high n-propanol-producing bacteria

The sample X characterized by its high n-propanol content in XQBJ was selected and inoculated into 150 mL of MRS, SS, and SMRS liquid triangular flask media at a 5% (w/w) inoculation rate. Static cultures were maintained at 30°C for 5 days. The sample with the highest n-propanol content was serially diluted and plated onto SMRS agar plates containing 100 mg/L of nystatin. Plates were incubated at 30°C for 3 days, and single colony morphology was observed. Forty single bacterial colonies with distinct morphological characteristics were selected for 16S rRNA gene sequence analysis and strain identification. Twenty bacterial strains representing different species were selected and inoculated into 250 mL Erlenmeyer flasks containing 150 mL of SMRS medium. Static cultures were maintained at 30°C for 7 days. The strain exhibiting the highest n-propanol production ability was selected, and its morphological characteristics were captured using scanning electron microscopy.

### 2.5 Establishment of an absolute quantitative method for *L. diolivorans*

Total DNA was extracted from fermented grains following the method described in the literature (Liu et al., 2017). The genome sequence of *L. diolivorans* (GenBank GCA_007992335.1) was obtained from the National Center for Biotechnology Information (NCBI) database. Species-specific primers for *L. diolivorans* were designed based on the propanediol dehydratase large subunit *pduC* gene (GenBank Accession No. WP 004105132.1). We designed one primer to target a DNA sequence encoding a species-specific loop region absent in orthologous proteins, and positioned the second primer in a nearby low-similarity region to maintain the efficiency of the qPCR process. This dual strategy, exploiting both structural divergence and sequence dissimilarity, maximized specificity and amplification robustness. PCR was used to validate primer specificity using genomic DNA from the target organism and non-target organisms as templates (Du et al., 2020). A standard curve was generated as previously described in the literature (Du et al., 2020).

### 2.6 Isolation of LAB and rapid identification of *L. diolivorans* from SFBJ fermented grains

Two fermented grain samples exhibiting the highest n-propanol content after 7–14 days of bottle pit fermentation were selected from five stacked material samples for LAB separation. Following serial dilution of the fermented grains, LAB colonies were isolated by plating appropriate dilutions onto SMRS agar plates containing nystatin. Plates were incubated at 30°C for 3 days, and 120 colonies with distinct colony morphologies from different dilutions were selected and purified through repeated streaking. These purified colonies were numbered and used for rapid *L. diolivorans* identification and systematic bacterial classification.

For rapid *L. diolivorans* identification, the Ezup Column Bacteria Genomic DNA Purification Kit (Sangon Biotech, Shanghai, China) was used to extract DNA. Following PCR amplification with specific primers, detection was performed using 1% agarose gel electrophoresis and subsequent observation with an automated gel imaging analysis system.

### 2.7 Characterization of *L. diolivorans* ZX6

For seed cultures, *L. diolivorans* ZX6 was grown in MRS medium under static conditions at 30°C for 72 h. Then, different fermentation media were inoculated at 2% (v/v) for the study. The growth and metabolic curves of *L. diolivorans* ZX6 were measured in MRS and SMRS media under static culture conditions for 10 days. MRS media with varying pH values (3.5, 4.0, 4.5, 5.0, 5.5, 6.0, and 6.5) were adjusted using a mixed acid solution (lactic acid: acetic acid = 1:1) and sodium hydroxide. To investigate the effects of pH and temperature on *L. diolivorans* ZX6 growth, experiments were conducted in an MRS medium at different pH or temperatures (20, 25, 28, 30, 32, 34, 37, 40, and 42°C) under static culture conditions at 30°C for 24 h. The impact of shaker speed (0, 50, 180 rpm) on n-propanol production by *L. diolivorans* ZX6 was evaluated in SMRS medium at 30°C for 7 days.

MRS media containing varying glucose concentrations (2%, 5%, 9%, 12%, 15%, 20%, 30%, and 35% w/v) were used to assess the impact of glucose concentration on n-propanol production by *L. diolivorans* ZX6. Fermentation experimental media with different carbon sources were prepared by replacing glucose in MRS medium with an equivalent amount (5% w/v) of sucrose, fructose, and xylose, respectively. All fermentations were conducted under static culture conditions at 30°C for 7 days.

### 2.8 RNA extraction, sequencing, and analysis

*L. diolivorans* ZX6 was inoculated (2% v/v) into MRS medium containing individual sugars (5% w/v each of glucose, sucrose, fructose, and xylose) as the sole carbon sources. Fifty milliliters of each culture were collected during the n-propanol index production period (5 d) and centrifuged to obtain the sediment, frozen in liquid nitrogen for 30 min, and immediately transferred to −80°C for storage. Three independent repeat experiments were conducted for each treatment group. RNA extraction, quantification, and sequencing were performed by Shanghai Majorbio Bio-pharm Biotechnology Co., Ltd. (Shanghai, China).

The high-quality sequencing data were mapped to the *L. diolivorans* DSM 14421 genome (GenBank: GCA_001434255.1), and the original data were uploaded to NCBI (BioProject: PRJNA1225471). The expression level and quantify abundances of each gene were calculated based on previous literature (Chen et al., 2023). The pathways and enzymes of flavor metabolism related to n-propanol were obtained from the KEGG database and previous literature (Gao et al., 2024).

### 2.9 Physical and chemical analysis

To analyze higher alcohol, ethanol, and acetic acid content in fermented broth or fermented grains, 50 g or 50 mL samples were subjected to pretreatment. Distillation was employed as a pretreatment method to quantify these compounds (Cao et al., 2010). Higher alcohols, ethanol, and acetic acid were analyzed using an Agilent 7820 gas chromatograph equipped with flame ionization detection (Agilent, USA) (López-Vázquez et al., 2010). A J&W CP-Wax 57 CB Acidic column (50 m × 0.25 mm × 0.2 μm, Agilent, USA) was used.

To analyze propionic acid and lactic acid concentrations, fermentation broths were centrifuged at 12,000 rpm for 10 min to remove cells. The supernatant was filtered through a 0.22 μm syringe filter and mixed with methanol in a 1:2 ratio. The mixture was shaken and stored at −20°C overnight to precipitate proteins and polysaccharides. Following this incubation period, the solution was centrifuged at 12,000 rpm for 10 min, filtered through a 0.22 μm syringe filter, and transferred to a liquid phase bottle for detection. High-performance liquid chromatography analysis was performed using an Agilent 1260 Infinity II with an Agilent C18 column, following the method described in a previous study (Sugiura et al., 2023). The peak areas of standard samples were used to determine propionic acid and lactic acid concentrations in the fermentation samples.

Reducing sugars were assayed using the 3,5-dinitrosalicylic acid method (Solarbio, Beijing, China). pH was measured using a pH meter.

### 2.10 Statistical analysis

Data were reported as mean ± standard deviation (SD) based on three independent determinations of each sample. Statistical analyses were performed using Microsoft Excel for Windows. Statistical significance was considered at *P* < 0.05, and results were expressed as mean ± SD.

### 2.11 Data availability

The raw sequence data of the 16S rRNA amplicon sequencing have been deposited in NCBI with the accession numbers PQ136908, PQ136909, PQ136910, and PQ136911.

## 3 Results

### 3.1 Enrichment and isolation of high n-propanol-producing bacteria

Considering the convenience of sampling, we initially selected XQBJ fermented grains with high n-propanol content (sample X) as the material for enrichment culture. Sample X was added to liquid culture media, including MRS, SS, and SMRS, for enrichment cultivation. Subsequently, samples were analyzed for n-propanol content. As shown in Table 1, n-propanol content was relatively low when fermented with MRS and SS media. However, when fermented with SMRS mixed media, the n-propanol content increased significantly to 326.60 mg/L, which was 5.9 and 12.6 times higher than that of MRS and SS media, respectively. This suggests that SMRS medium could effectively enriches microorganisms with high n-propanol production capabilities. The n-propanol content of sample X-N4, supplemented with antifungal nystatin, reached 431.70 mg/L. In contrast, n-propanol was not detected in sample X-N5, supplemented with antibacterial ampicillin. These results indicated that the n-propanol-producing microorganisms in the SMRS fermentation broth were bacteria.

**Table 1:** Contents of main compounds after inoculation of fermented grain from XQBJ in different fermentation media (mg/L).

**No**.	**Fermentation media**	**Ethanol (%vol)**	**Isobutanol**	**Isoamylol**	**Phenylethanol**	**n-Propanol**	**Acetic acid**
X-N1	MRS	0.14 ± 0.02	ND^a^	ND	ND	55.00 ± 3.45	330.5 ± 22.03
X-N2	SS	2.74 ± 0.50	32.9 ± 4.45	59.6 ± 3.54	15.6 ± 2.14	26.01 ± 4.51	3,465.0 ± 123.87
X-N3	SMRS	0.60 ± 0.04	ND	13.81 ± 2.54	ND	326.60 ± 24.14	2,595.3 ± 103.59
X-N4	SMRS + nystatin	0.11 ± 0.03	ND	ND	ND	431.70 ± 14.51	4,986.0 ± 221.45
X-N5	SMRS + ampicillin	0.31 ± 0.04	ND	32.4 ± 2.67	20.9 ± 1.10	ND	6,301.4 ± 295.14

^a^ND means not detected.

**Figure 1:**
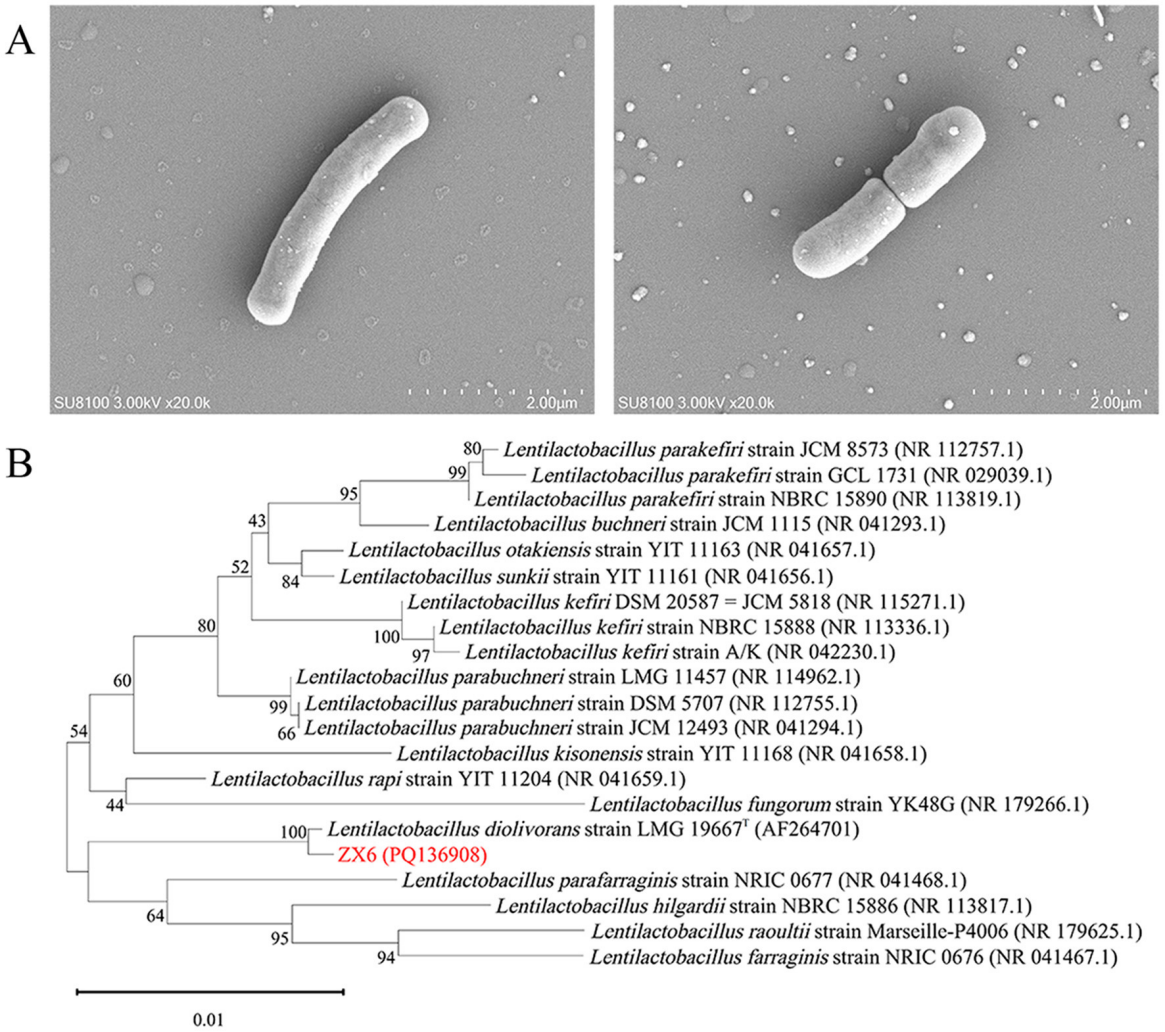
Identification of *L. diolivorans* ZX6. **(A)** The morphology of *L. diolivorans* ZX6 by SEM analysis, **(B)** phylogenetic tree of *L. diolivorans* ZX6 based on 16S rRNA gene sequences.

To isolate n-propanol-producing bacteria from sample X-N4, 40 bacterial colonies were selected based on morphological differences and purified through repeated streaking on SMRS plates. These strains, named ZX1 to ZX40, were classified into 9 species of LAB and one species of *Acetobacter pasteurianus* based on 16S rRNA gene sequence homology analyses. Twenty bacterial strains representing distinct species were inoculated into SMRS liquid medium to evaluate their n-propanol production capacity through fermentation experiments. Among these, strain ZX6 demonstrated the highest n-propanol yield, reaching 3,499.40 mg/L after a 7-day fermentation (Supplementary Table S2). No n-propanol was detected in the fermentation broth of the other 19 bacterial strains. Strain ZX6 was rod-shaped and Gram-positive. Its cell morphology was examined using scanning electron microscopy (Figure 1A). The 16S rRNA gene of strain ZX6 was amplified, resulting in a 1,450 bp single fragment that was subsequently sequenced (GenBank Accession No. PQ136908). BLAST analysis revealed a high similarity (>99.7%) between the ZX6 sequence and the 16S rRNA gene sequence of *L. diolivorans* LMG 19667^T^ (AF264701) (Krooneman et al., 2002). A phylogenetic tree was constructed (Figure 1B). Based on colony morphology, morphological characteristics, and 16S rRNA gene sequence analysis, strain ZX6 was identified as *L. diolivorans*.

To evaluate the n-propanol production capability of *L. diolivorans* under brewing conditions, co-culture experiments with *S. cerevisiae* were conducted. Both liquid co-culture results (Supplementary Figure S1A) and solid-state co-culture results (Supplementary Figure S1B) of *L. diolivorans* ZX6 and *S. cerevisiae* SCY62 demonstrated a direct positive correlation between n-propanol content and the initial inoculation amount of *L. diolivorans* ZX6. When *L. diolivorans* ZX6 and *S. cerevisiae* SCY62 were mixed in a 1:1 (v/v) ratio, the n-propanol content in the SMRS fermentation broth was 246.01 mg/L. During solid-state fermentation, with an *L. diolivorans* ZX6 inoculation amount of 10^6^ CFU/g, the n-propanol content in the fermented grain reached 1,762.05 mg/kg. Under solid-state fermentation conditions, high inoculation of *L. diolivorans* resulted in n-propanol content in laboratory-scale fermented grains equivalent to that in the first round of fermented grains from SFBJ distillery.

### 3.2 Absolute quantification of *L. diolivorans* in SFBJ fermented grains

Next, qPCR was conducted to quantify *L. diolivorans* in microbiome samples. Species-specific primers for *L. diolivorans* were designed: F: 5′-TTTCATTTGGTAAGAAGGATTCTTCTG-3′ and R: 5′-TAATATCACCATTTCCTGATGTGGAC-3′. These primers exhibited 100% exclusivity for the other 12 non-target microorganisms from Baijiu fermented grains (Supplementary Table S3), with no matches to other organisms in the NCBI database. We subsequently developed a qPCR method using these specific primers. The standard curve obtained was y = −3.4462x + 46.017 (*R*^2^ = 0.99), demonstrating a linear correlation (*R*^2^ = 0.99) over a range of 65.47 to 6.547 × 108 copies per reaction. This suggests that the qPCR method is suitable for quantifying *L. diolivorans* in the samples.

**Figure 2:**
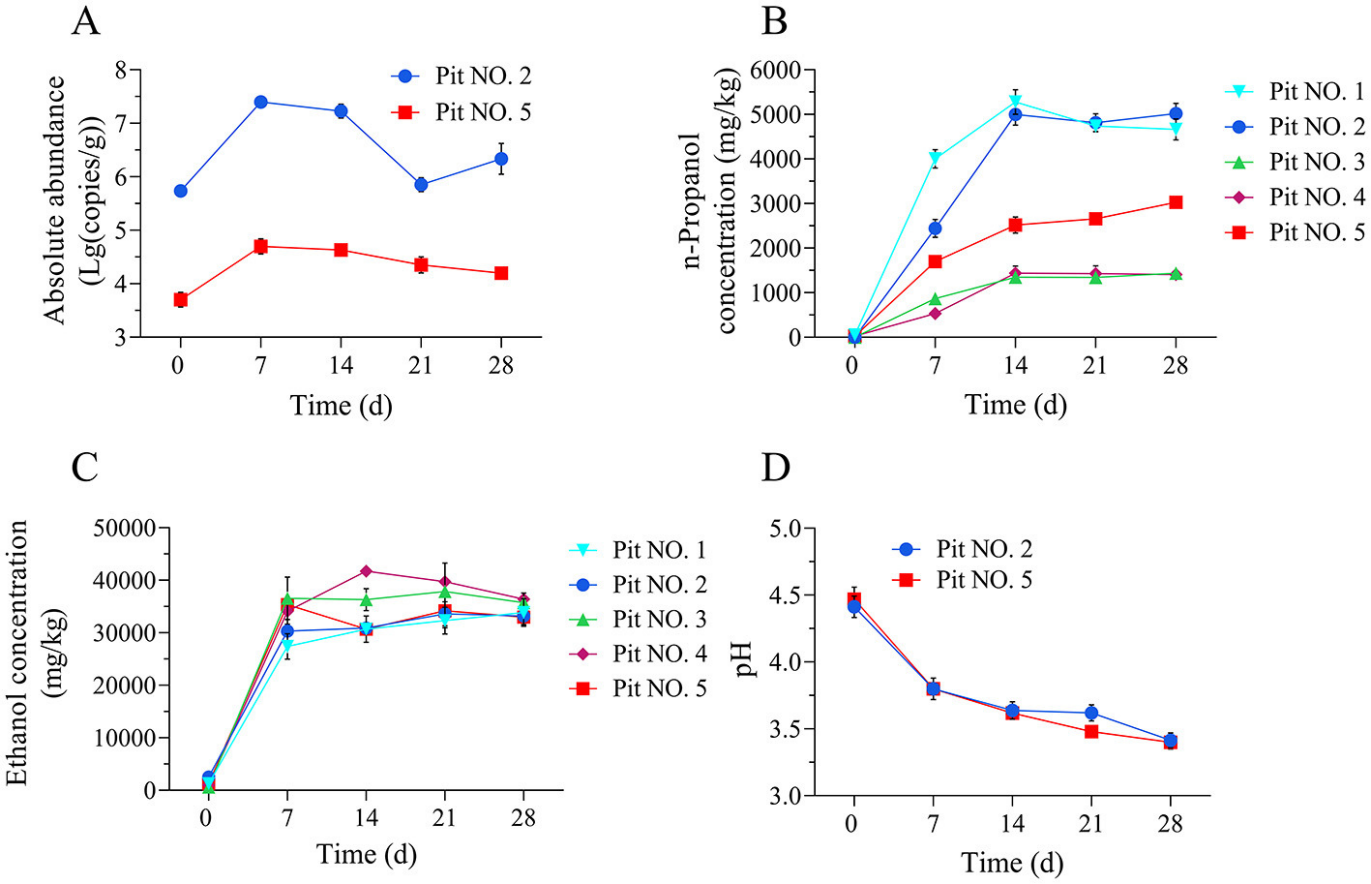
Changes in the quantities of *L. diolivorans* and the n-propanol content in different fermented grains during pit fermentation. **(A)** The absolute abundance of internal standard *L. diolivorans* in fermented grains of Pit No. 2 and Pit No. 5, **(B)** n-propanol content, **(C)** ethanol content, and **(D)** pH.

Significant differences were observed in the n-propanol content of the five fermented grains from the scaled-down pit fermentation experiment (Figure 2B). Sample No. 2, characterized by its high n-propanol content, was selected for the *L. diolivorans* bacterial count determination using qPCR. Initial *L. diolivorans* quantities were relatively low, at 5.51 × 10^5^ copies/g, but rapidly increased to 2.50 × 107 copies/g by the 7th day of fermentation. Subsequently, the bacterial count decreased significantly, reaching 7.08 × 105 copies/g at the end of fermentation (Figure 2A). The quantities of *L. diolivorans* in sample No. 5 (which had an n-propanol content in the middle range) showed a similar trend, reaching a maximum of 4.98 × 10^4^ copies/g on the 7th day and then gradually decreasing throughout the fermentation process. This decline might be attributed to the accumulation of acidity and alcohol content in the later stages of fermentation, which inhibits *L. diolivorans* growth. n-Propanol and ethanol concentrations in the fermented mash increased rapidly in the early stages of fermentation, reaching their peak by the 14th day and remaining relatively constant thereafter (Figures 2B, C). The pH of the fermented grains decreased significantly during the first 2 weeks of fermentation, from around pH 4.5 to pH 3.7, and then stabilized in the last 2 weeks (Figure 2D). Changes in the *L. diolivorans* bacterial count within the fermented grains generally aligned with the increasing trend of n-propanol content. However, the n-propanol content typically reached its peak around the 14th day of fermentation, exhibiting a slight lag compared to the peak *L. diolivorans* count.

### 3.3 Rapid identification of *L. diolivorans* from SFBJ fermented grains

One hundred and seven LAB strains, numbered ZD1 to ZD107, were isolated and purified from SFBJ fermented grains with high n-propanol content. Three strains with positive gel electrophoresis bands were identified: ZD9 (GenBank Accession No. PQ136909), ZD92 (GenBank Accession No. PQ136910), and ZD93 (GenBank Accession No. PQ136911). BLAST analysis revealed that these three strains shared high similarity (>99.7%) with the 16S rRNA gene sequence of *L. diolivorans* LMG 19667^T^ (AF264701), confirming their classification as *L. diolivorans*. This demonstrated the high reliability of the specific primer method for rapid *L. diolivorans* identification. 16S rRNA gene sequence analysis and homology alignment classified the 107 LAB strains into 7 genera and 12 species (Supplementary Figure S2A). The three most prevalent LAB species were *L. buchneri, L. panis*, and *Lactobacillus pontis*, accounting for 57.94%, 12.15%, and 10.28%, respectively. The other 9 species accounted for < 5%, and *L. diolivorans* accounted for only 2.80% (Supplementary Figure S2B). Fermentation tests of the three *L. diolivorans* strains from SFBJ fermented grain revealed n-propanol contents exceeding 2,400 mg/L, similar to *L. diolivorans* ZX6. However, no significant n-propanol production was detected in samples fermented by other LAB (Supplementary Table S4). This indicated that *L. diolivorans* was the primary microorganism isolated from SFBJ fermented grains with the capacity to synthesize high concentrations of n-propanol.

### 3.4 Characterization of *L. diolivorans* ZX6

#### 3.4.1 Effects of different media and cultivation time on L. diolivorans ZX6

**Figure 3:**
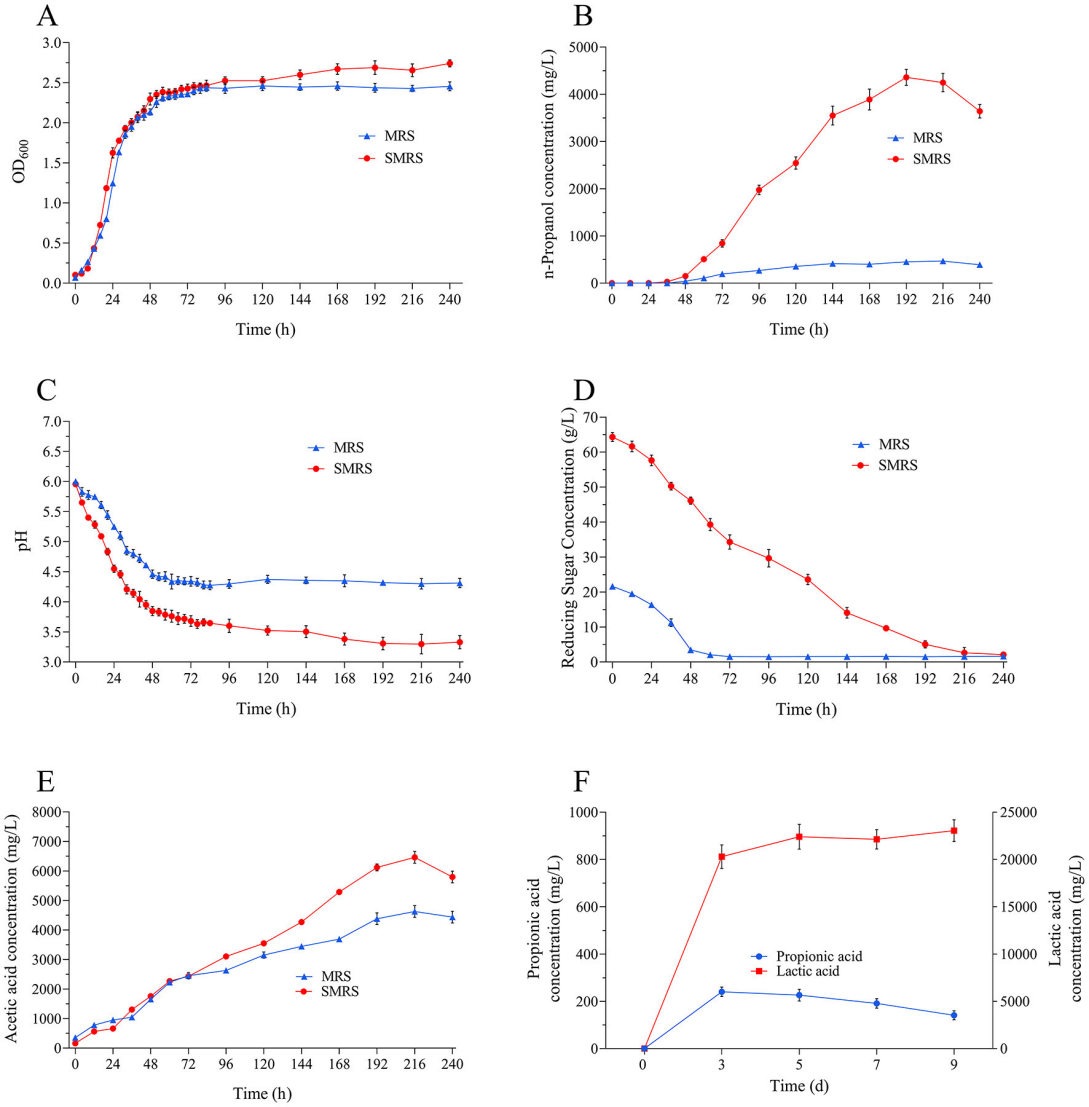
Growth curves and fermentation analysis of *L. diolivorans* ZX6 in different media. **(A)** OD_600_, **(B)** n-propanol, **(C)** pH, **(D)** reducing sugar, **(E)** acetic acid, **(F)** propionic acid and lactic acid in SMRS medium.

Two media, MRS and SMRS, were used to investigate the effects on *L. diolivorans* ZX6 growth and metabolism. The OD_600_ of *L. diolivorans* ZX6 in the SMRS medium was slightly higher than that in the MRS medium (Figure 3A). However, a significant difference was observed in n-propanol production during fermentation (Figure 3B). The n-propanol content in both media increased significantly after 48 h of fermentation. In SMRS, it reached a maximum of 4,399 mg/L at 192 h, while in MRS, it reached a maximum of 472 mg/L at 216 h. The decreasing trend of reducing sugars in the media aligned with the increasing n-propanol content (Figure 3D). The pH of both culture media rapidly decreased within the first 48 h of fermentation, from the initial pH of 6.0 to below pH 4.5. While the pH of MRS fermentation broth stabilized around pH 4.5, the pH of SMRS fermentation broth continued to decrease to around pH 3.5 (Figure 3C). After 216 h of fermentation, the acetic acid content peaked at 4,000 mg/L in MRS and 6,000 mg/L in SMRS (Figure 3E).

In the SMRS medium, lactic acid content increased rapidly during the early stages of fermentation. This growth pattern closely aligned with biomass accumulation, with lactic acid content reaching 23,053.5 mg/L after 9 days. Conversely, propionic acid content remained very low at 144.6 mg/L (Figure 3F).

#### 3.4.2 Effects of fermentation conditions on *L. diolivorans* ZX6

**Figure 4:**
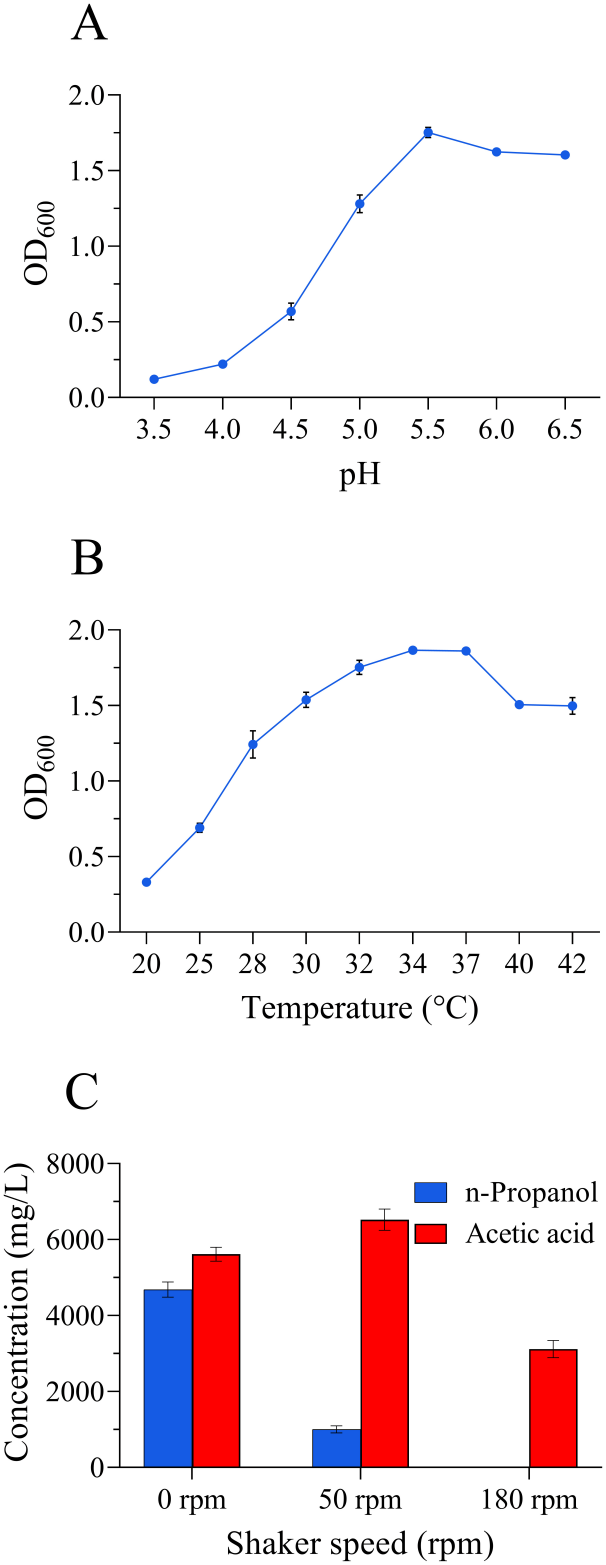
Effects of pH, temperature, and shaking speed on the growth of *L. diolivorans* ZX6. **(A)** pH, **(B)** temperature, and **(C)** shaker speed.

The optimal growth pH of *L. diolivorans* ZX6 was 5.5, which aligned with the standard strain *L. diolivorans* LMG 19667^T^ (Figure 4A). The optimal growth temperature of *L. diolivorans* ZX6 was 34°C (Figure 4B), which is similar to that of the LMG 19667^T^ strain. However, *L. diolivorans* ZX6 strain exhibited greater heat resistance than the LMG 19667^T^ strain. Fermentation experiments conducted at various shaking speeds revealed significantly higher n-propanol production by *L. diolivorans* ZX6 during static fermentation compared to shaking cultivation (Figure 4C). This suggests that *L. diolivorans* is more sensitive to oxygen during n-propanol synthesis.

#### 3.4.3 Effects of carbon source types and glucose concentration on *L. diolivorans* ZX6

The effect of glucose concentration on n-propanol content demonstrated that a 5% glucose concentration in MRS medium yielded the maximum n-propanol content of 1,555.9 mg/L. As the glucose concentration increased, n-propanol yield gradually decreased (Supplementary Figure S3).

**Figure 5:**
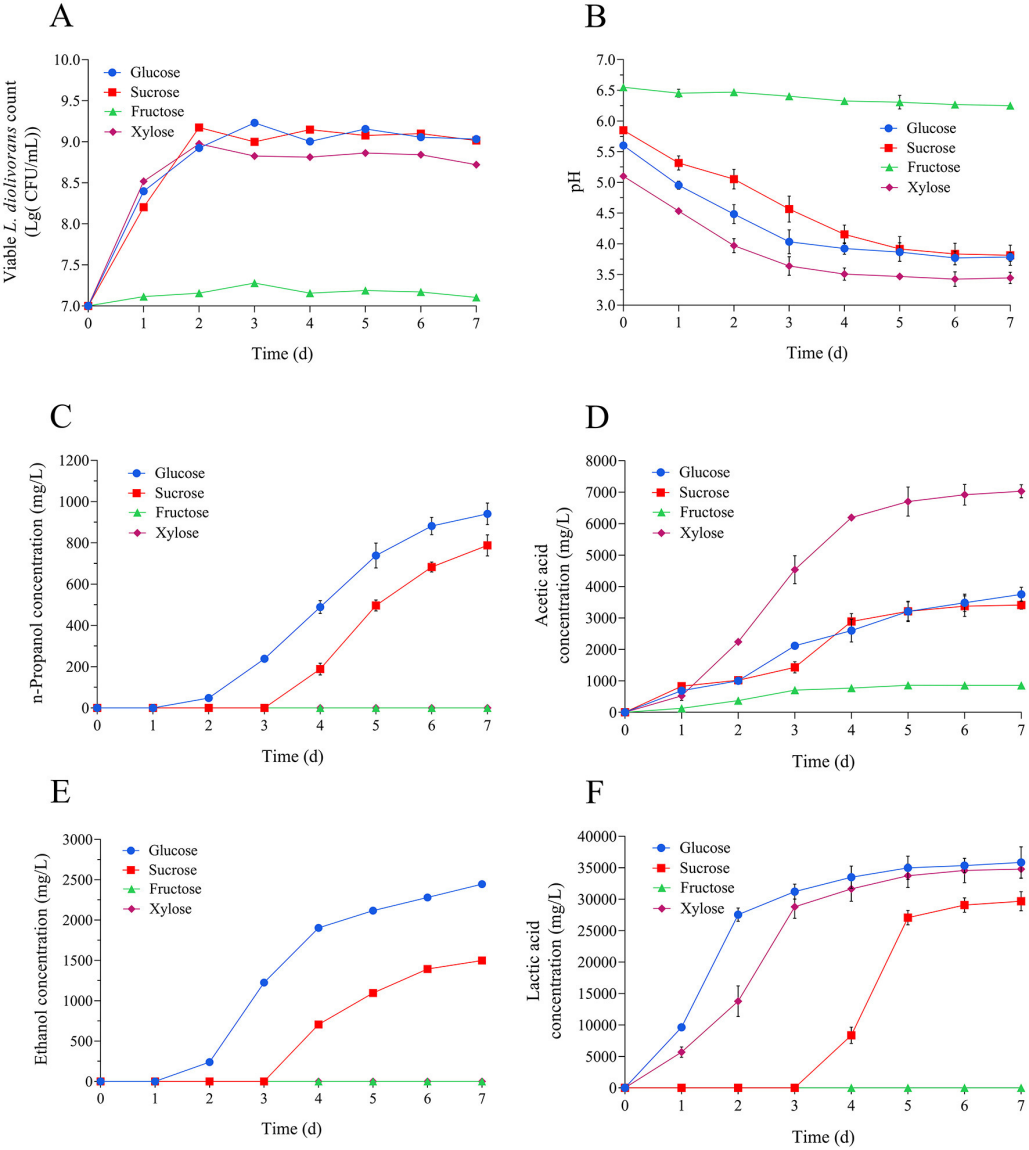
Effects of different carbon sources on the growth and metabolite production of *L. diolivorans* ZX6. **(A)** Viable cell counts, **(B)** pH, **(C)** n-propanol, **(D)** acetic acid, **(E)** ethanol, and **(F)** lactic acid.

*L. diolivorans* ZX6 demonstrated substantial biomass accumulation during cultivation in MRS medium containing glucose, xylose, or sucrose as the carbon sources. In contrast, biomass production decreased with fructose as the sole carbon source (Figure 5A). Fermentation with glucose, xylose, or sucrose as carbon sources led to progressive pH reduction, whereas fructose utilization resulted in minimal pH variation (Figure 5B). Cultures utilizing glucose or sucrose synthesized multiple metabolites, including n-propanol (Figure 5C), acetic acid (Figure 5D), ethanol (Figure 5E), and lactic acid (Figure 5F). Xylose-fed cultures, however, generated exclusively lactic acid and acetic acid.

#### 3.4.4 Comparative analysis of transcriptome during fermentation of different carbon sources

**Figure 6:**
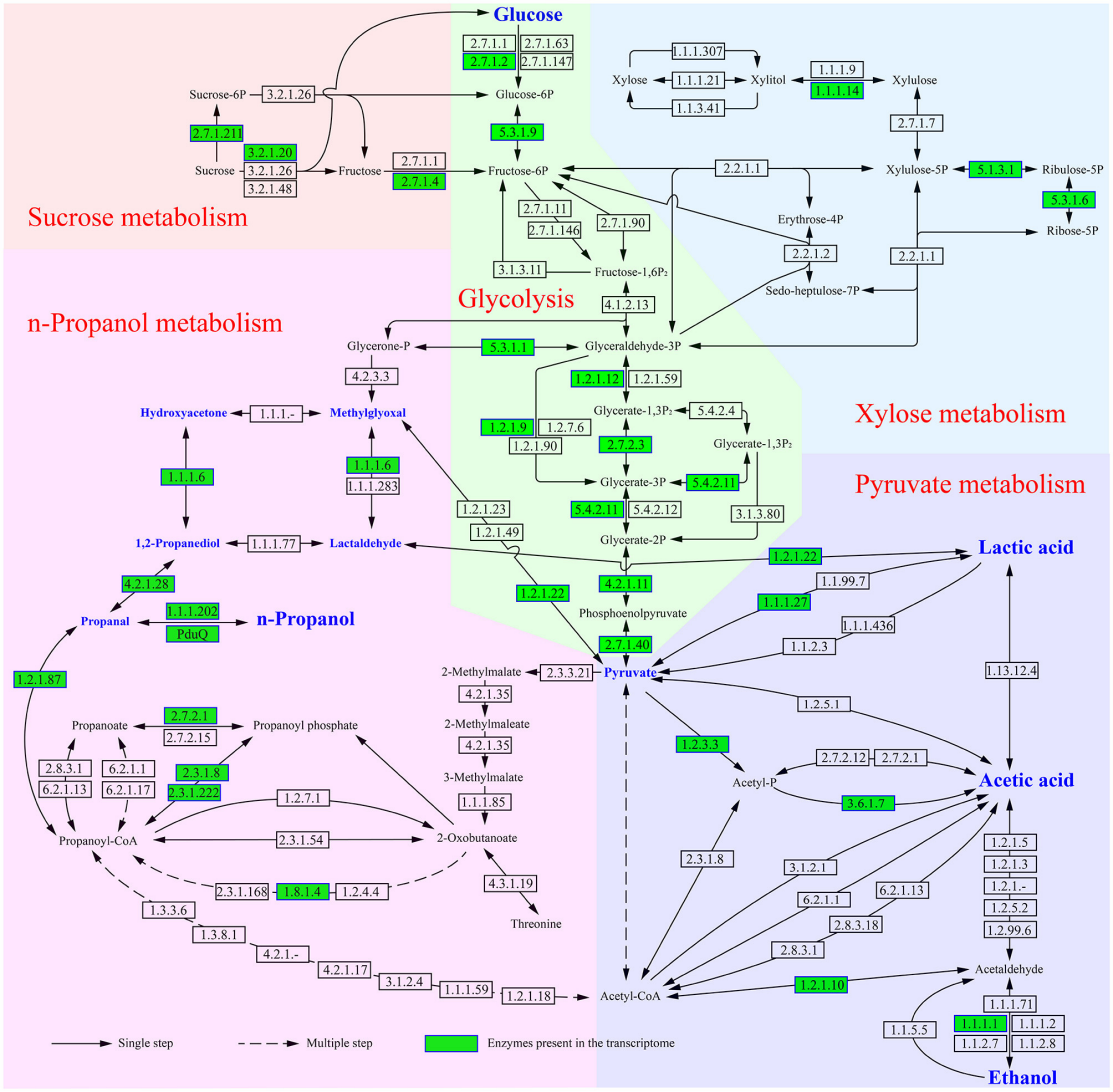
Metabolic pathways and enzymes involved in the conversion of four carbon sources into n-propanol, lactic acid, ethanol, and acetic acid.

**Figure 7:**
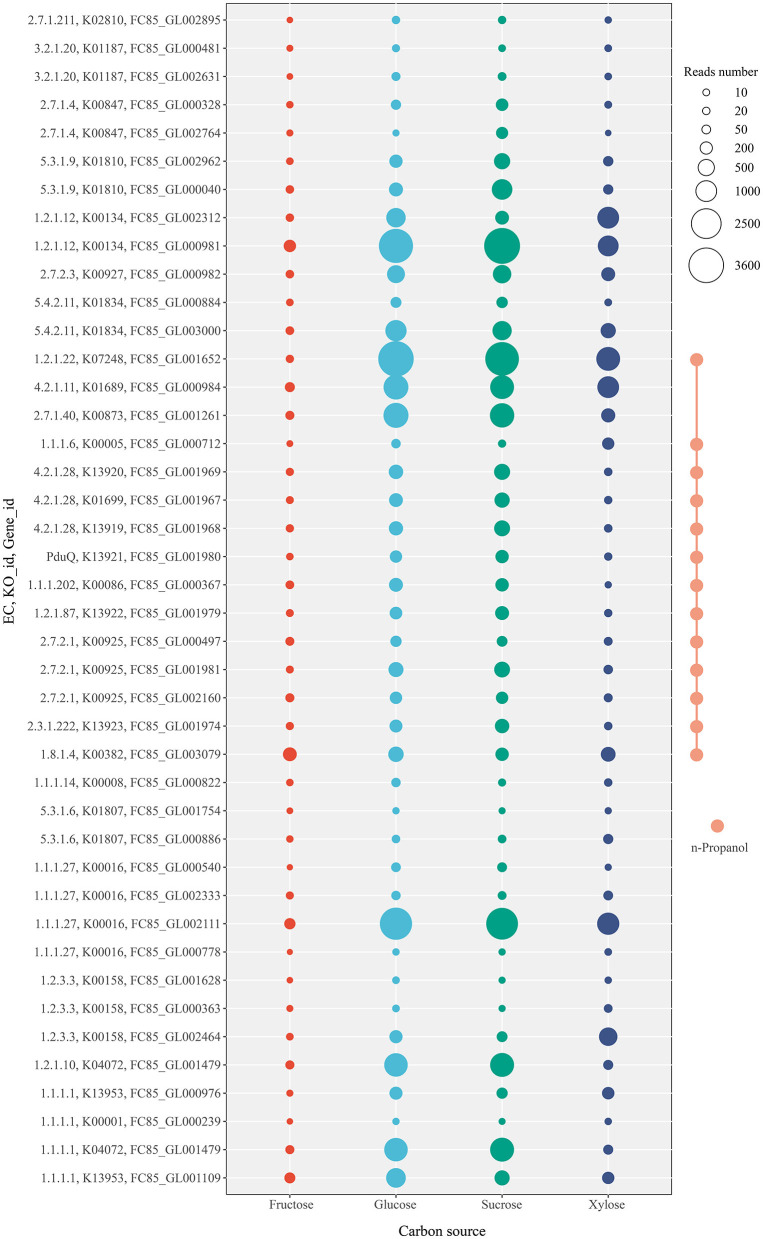
Transcriptional levels of genes in *L. diolivorans* ZX6 cultured with four different carbon sources.

When *L. diolivorans* ZX6 was cultured with four different carbon sources, the transcriptome analysis was performed on the 5th day of the rapid growth period of n-propanol. Based on the KEGG database and previous literature, we mapped the metabolic pathways and associated enzymes involved in the metabolism of n-propanol, lactic acid, ethanol, and acetic acid (Figure 6). A total of 94 enzymes were involved in the metabolic pathway, with 30 enzymes detected in the transcriptome. Among these, 24 enzymes exhibited significant differences in transcription levels, and 42 genes were associated with these enzymes (Supplementary Figure S4). Transcriptome data indicated that n-propanol is likely formed through the methylglyoxal pathway, in which methylglyoxal is converted to propanal via 1,2-propanediol and subsequently reduced to n-propanol. Three precursors entered the methylglyoxal pathway: glycerone-P, pyruvate, and lactic acid. Since methylglyoxal synthase (EC 4.2.3.3) was not present in the transcriptome and triosephosphate isomerase (EC 5.3.1.1) did not show significant differences in transcription levels across different carbon sources, glycerone-P was likely not the primary precursor for *L. diolivorans* ZX6 to enter the methylglyoxal pathway. Lactaldehyde dehydrogenase (EC 1.2.1.22) catalyzed the formation of lactaldehyde from lactic acid and the conversion of pyruvate to methylglyoxal. Additionally, the transcriptional level of its gene was significantly higher in cultures with glucose and sucrose than in those with fructose and xylose (*P* ≤ 0.001) (Figure 7 and Supplementary Figure S4, C1). Similar results were observed for the transcription levels of the three enzymes (EC 4.2.1.28, 1.1.1.102, and PduQ) involved in the conversion of 1,2-propanediol to n-propanol (Supplementary Figure S4 C5, C6, D1– D3). The transcriptional levels were consistent with the increase in n-propanol content: compared to cultures with fructose and xylose as carbon sources, n-propanol production significantly increased by day 5 when *L. diolivorans* ZX6 was cultured with glucose and sucrose as carbon sources (Figure 5C). Lactate dehydrogenase (EC 1.1.1.27) (Supplementary Figure S4, F1–F4) and pyruvate oxidase (EC 1.2.3.3) (Supplementary Figure S4, F5, F6, G1) were inferred as the key enzymes for *L. diolivorans* ZX6 to metabolize pyruvate into lactic acid and acetic acid, respectively.

## 4 Discussion

The mechanism underlying high n-propanol production in Baijiu brewing, particularly in SFBJ, has long been an industry enigma. In this study, a high n-propanol-producing strain of *L. diolivorans* was isolated from Baijiu fermented grains, which may be a key bacterium responsible for high n-propanol formation during Baijiu brewing. *L. diolivorans* has not been previously detected or reported in Baijiu fermentation systems, primarily due to its low abundance in Baijiu fermented grains. Even in SFBJ fermented grains with high n-propanol concentrations, *L. diolivorans* constitutes only about 2.8% of the total easily culturable LAB on aerobic plates (Supplementary Figure S2B), making it susceptible to dilution and potential exclusion from conventional culture methods. Moreover, the similar colony morphology of *L. diolivorans* to dominant LAB species like *L. buchneri* increases the likelihood of overlooking high n-propanol-producing strains during random colony selection. The complex microbial community in Baijiu fermentation, coupled with the diversity of species and n-propanol-producing microbial synthetic pathways (Pang et al., 2018; Du et al., 2019; Hao et al., 2021; Zhang et al., 2021), significantly complicates tracing the causal relationship between n-propanol and its producing microbial species (Liu and Miao, 2020; Xiao et al., 2021; Luo et al., 2023).

In addition, research on the microbial sources of high n-propanol production in SFBJ is constrained by the fermented grain materials. The fermentation process of SFBJ is not continuous; it involves a crucial preliminary solid-state stacking stage prior to pit fermentation (Chen et al., 2024a). Each round of fermented grains undergoes ~30 days of pit fermentation. Specifically, the fermentation and sampling period for the first round of fermented grains from the Baijiu distillery with the highest n-propanol content is limited to ~1 month annually. This limitation hinders the progression and deepening of research on the source of high concentrations of n-propanol in Baijiu. In contrast to SFBJ, the fermentation of XQBJ does not necessitate a high-temperature stacking process, and the fermentation period is relatively short (~10 days), making it convenient for factory sampling and small-scale fermentation experiments in the laboratory. More importantly, a significant amount of n-propanol is produced during the brewing process of XQBJ. Consequently, XQBJ fermented grains were selected as the material for enrichment culture and solid-state fermentation, thereby overcoming the limitations imposed by material availability and the challenges associated with conducting repeatable solid-state fermentation experiments of Baijiu at the laboratory scale. Furthermore, the *L. diolivorans* ZX6 strain, characterized by its high n-propanol production capacity, was successfully isolated from XQBJ fermented grains for the first time. Compared to MRS medium, the use of SMRS medium for the enrichment culture of XQBJ fermented grains and for fermentation research on *L. diolivorans* ZX6 can lead to a higher production of n-propanol. One contributing factor may be the increased glucose concentration. The specific reasons for this influence still require more detailed research in the future.

qPCR analysis revealed *L. diolivorans* counts ranging from 1.29 × 104 to 3.30 × 105 copies/g in fermented grains from three distilleries (Supplementary Table S1). This indicates *L. diolivorans* is widely distributed in SFBJ mash across different regional manufacturers. To our knowledge, this is the highest-yielding natural microorganism reported for fermentative n-propanol production directly from glucose. Furthermore, when *L. diolivorans* was co-cultured with *Rhizopus* and *S. cerevisiae* in solid-state fermentation, the n-propanol yield also reached the highest level reported to date. Therefore, its presence in fermented grains may be the main reason for the production of high n-propanol levels in Baijiu. Due to the limited number of samples collected, further in-depth research is required to investigate the origin of *L. diolivorans*, its population dynamics during Baijiu brewing, and the differences in n-propanol production capacity among strains from different regions.

Nutritional and environmental factors in Baijiu brewing process, such as the carbon-to-nitrogen ratio have important influence on n-propanol content (Jiang et al., 2019). Our research results show that high n-propanol synthesis from *L. diolivorans* ZX6 required high sugar concentrations (Supplementary Figure S3). Reducing sugar, acidity, alcohol, and temperature have been established as the primary driving forces in stacking and pit fermentation (Hao et al., 2021). In the present study, *L. diolivorans* abundance increased rapidly only in the early fermented grain, possibly due to its weak acid resistance. The optimal pH for *L. diolivorans* ZX6 was 5.5, and growth slowed below pH 4.0 (Figure 4A). This aligned with the detection of increasing acidity and decreasing n-propanol content in fermented grains with advancing fermentation rounds in SFBJ distilleries (Guo et al., 2022). As acidity increased, LAB with stronger acid resistance, such as *L. acetotolerans* and *Acetilactobacillus jinshanensis*, became dominant in the middle and later stages of fermented grains, replacing weaker acid-resistant LAB like *L. diolivorans* (Du et al., 2020; Liao et al., 2023).

*L. diolivorans* has been reported to coexist with various LAB in specific traditional fermented foods, including French sourdoughs (Lhomme et al., 2015), Mongolian fermented dairy products (Yu et al., 2011; Sánchez-Juanes et al., 2020), and other acidic fermented foods. This coexistence reflects the general adaptability of LAB to acidic growth environments. In silage feed and sourdough, *L. diolivorans* can utilize 1,2-propanediol, a metabolic product of *L. buchneri*, to synthesize propionic acid and n-propanol (Krooneman et al., 2002; Zhang et al., 2010). Our results demonstrated that *L. diolivorans* ZX6 possesses a complete metabolic pathway to metabolize glucose into n-propanol, lactic acid, acetic acid, and ethanol. Although transcriptome data suggest that n-propanol is likely formed through the methylglyoxal pathway, further validation through knockout mutant studies and overexpression studies remains to be explored. When fermenting with glucose as a substrate, *L. diolivorans* ZX6 produces substantial amounts of n-propanol. Is there a synergistic effect between the coexistence of *L. diolivorans* and *L. buchneri* in SFBJ fermented grains on the production of n-propanol? Follow-up research is needed. Additionally, the interactions of *L. diolivorans* with other dominant microbes, such as yeasts and LAB, within solid-state fermented grains, as well as their impact on n-propanol content and other flavor substances in Baijiu, are topics requiring in-depth exploration.

## 5 Conclusion

In conclusion, we isolated a rare *L. diolivorans* strain, ZX6, from Baijiu fermented grains. The pure strain exhibited n-propanol fermentation yields exceeding 4,300 mg/L in SMRS medium. Utilizing the *pdu*C gene sequence of 1,2-propanediol dehydratase from *L. diolivorans*, we established a rapid PCR screening method for *L. diolivorans* and a qPCR method for its detection in fermented grains. *L. diolivorans* was detected in the first round of SFBJ fermented grains from three different regions, suggesting its potential role as one of the key microorganisms responsible for high n-propanol production. *L. diolivorans* ZX6 directly metabolized glucose to produce substantial amounts of n-propanol. High n-propanol synthesis occurred under high sugar concentrations, anaerobic environments, and relatively long fermentation times. Transcriptome analysis revealed that *L. diolivorans* ZX6 likely synthesized 1,2-propanediol via the methylglyoxal pathway, which was subsequently converted to n-propanol. *L. diolivorans* coexisted with the dominant *L. buchneri* in SFBJ fermented grains. Indeed, potential synergistic effects between these strains in n-propanol synthesis, as well as interactions between *L. diolivorans* and other dominant microorganisms, such as yeast and LAB, and their influence on n-propanol content, require further investigation. This study provides a theoretical foundation for developing processes to reduce n-propanol content in Baijiu brewing by artificially controlling the source and quantity of *L. diolivorans*.

## Data Availability

The datasets presented in this study can be found in online repositories. The names of the repository/repositories and accession number(s) can be found below: https://www.ncbi.nlm.nih.gov/, PRJNA1225471.
