# Supplementary material for: Isolation and characterization of Lentilactobacillus diolivorans: a high n-propanol-producing microorganism from Baijiu brewing
Source: Front Microbiol. 2025 Jun 25;16:1624097. doi: 10.3389/fmicb.2025.1624097 (PMC12238061; doi:10.3389/fmicb.2025.1624097)
Supplement: Supplementary file 1 [file Table_1.DOCX]

**Supplementary tables**

**Table S1** Contents of major flavor compounds and the absolute abundances of *L. diolivorans* in different SFBJ fermented grains from three distilleries.

| Sample Source | No. | n-Propanol  (mg/kg) | Isoamylol  (mg/kg) | Ethanol  (mg/kg) | Absolute abundances of  *L. diolivorans* (copies/g) |
| --- | --- | --- | --- | --- | --- |
| Songzi | D-1 | 2501.92±63.24 | ND^a^ | 15937.8±1893.6 | 101350.43 |
|  | D-2 | 2971.94±82.45 | ND | 15937.8±867.9 | 330128.59 |
| Maotai town | GZ-1 | 2294.14±19.25 | 12.15±1.23 | 22644.3±789 | 197257.29 |
|  | GZ-2 | 1657.94±44.47 | 13.82±0.28 | 21934.2±631.2 | 25193.88 |
| Shennongjia | SNJ-1 | 3090.87±72.68 | 13.79±0.35 | 36609.6±1183.5 | 91481.27 |
|  | SNJ-2 | 1684.41±50.04 | 12.65±0.14 | 29508.6±1262.4 | 12915.77 |

^a^ ND means not detected.

**Table S2** Contents of ethanol, n-propanol and acetic acid produced by LAB strains from XQBJ fermented grains.

| Strain | No. | Ethanol (%vol) | n-Propanol (mg/L) | Acetic acid (mg/L) |
| --- | --- | --- | --- | --- |
| *Lentilactobacillus buchneri* | ZX1 | 0.84±0.03 | ND | 1989.74±34.18 |
|  | ZX5 | 0.74±0.03 | ND | 2643.41±24.98 |
|  | ZX34 | 0.74±0.02 | ND | 2643.45±34.54 |
| *Lentilactobacillus diolivorans* | ZX6 | 0.94±0.02 | 3499.40±65.56 | 3844.94±55.15 |
| *Lactiplantibacillus plantarum* | ZX11 | 0.10±0.00 | ND | 804.41±11.88 |
|  | ZX20 | ND | ND | 1712.15±20.48 |
|  | ZX29 | 0.09±0.00 | ND | 1060.07±21.15 |
| *Lactobacillus helveticus* | ZX19 | ND | ND | 1821.92±31.47 |
|  | ZX30 | 0.10±0.00 | ND | 972.10±11.14 |
|  | ZX36 | 0.03±0.00 | ND | 1070.28±18.18 |
| *Levilactobacillus brevis* | ZX17 | 0.80±0.03 | ND | 1339.46±24.24 |
|  | ZX33 | 0.90±0.02 | ND | 1065.38±14.14 |
|  | ZX39 | 0.88±0.02 | ND | 1222.43±14.23 |
| *Lactobacillus harbinensis* | ZX32 | 0.21±0.02 | ND | 1058.40±19.97 |
| *Lactobacillus paracasei* | ZX10 | ND | ND | 1676.74±19.25 |
|  | ZX27 | 0.05±0.00 | ND | 1138.28±17.25 |
| *Acetobacter pasteurianus* | ZX2 | 0.09±0.01 | ND | 998.20±10.02 |
|  | ZX13 | 0.10±0.02 | ND | 1076.87±16.25 |
|  | ZX25 | 0.16±0.01 | ND | 1082.51±10.99 |
| *Lactobacillus parafarraginis* | ZX15 | 0.81±0.02 | ND | 3111.18±35.52 |

^a^ ND means not detected.

**Table S3** Primer-specific detections in various microorganisms.

| Strains | No. | *pdu*C | Strains | No. | *pdu*C |
| --- | --- | --- | --- | --- | --- |
| *Lentilactobacillus diolivorans* | ZX6 | +^a^ | *Lactobacillus hilgardii* | ZD42 | -^b^ |
| *Lentilactobacillus* *buchneri* | ZX1 | - | *Lacticaseibacillus rhamnosus* | ZD48 | - |
| *Limosilactobacillus panis* | ZD3 | - | *Lactobacillus amylovorus* | ZD13 | - |
| *Lactobacillus parafarraginis* | ZX15 | - | *Lactobacillus helveticus* | ZX19 | - |
| *Lactobacillus farraginis* | ZD83 | - | *Lactiplantibacillus plantarum* | ZX11 | - |
| *Lactobacillus pontis* | ZD8 | - | *Levilactobacillus brevis* | ZX33 | - |
| *Lactobacillus paracasei* | ZX10 | - |  |  |  |

^a^ +: *pdu*C present; ^b^ -: *pdu*C absent.

**Table S4** Contents of ethanol, n-propanol and acetic acid produced by LAB strains from SFBJ fermented grains.

| Strain | No. | Ethanol (%vol) | n-Propanol (mg/L) | Acetic acid (mg/L) |
| --- | --- | --- | --- | --- |
| *L. diolivorans* | ZD9 | 0.67±0.02 | 3430.34±70.81 | 5464.46±65.12 |
|  | ZD92 | 0.7±0.03 | 2461.43±58.77 | 3362.87±76.45 |
|  | ZD93 | 0.82±0.03 | 2616.36±53.51 | 4217.45±74.68 |
| *Limosilactobacillus panis* | ZD1 | 0.06±0.00 | ND^a^ | 2996.64±61.54 |
|  | ZD3 | 0.17±0.01 | ND | 1838.97±42.41 |
|  | ZD45 | 0.30±0.02 | ND | 2027.41±38.14 |
|  | ZD57 | 0.33±0.04 | ND | 2799.56±54.54 |
|  | ZD77 | 0.16±0.01 | ND | 2587.69±65.15 |
|  | CGMCC 1.3925 | 0.22±0.00 | ND | 2357.15±49.25 |
| *Lactobacillus pontis* | ZD8 | 0.21±0.05 | ND | 1429.37±30.04 |
|  | ZD15 | 0.31±0.01 | ND | 1644.05±34.91 |
| *Lactobacillus amylovorus* | ZD13 | 0.11±0.01 | ND | 1254.12±30.01 |
|  | ZD16 | 0.15±0.02 | ND | 1054.12±25.14 |
| *Lacticaseibacillus rhamnosus* | ZD48 | 0.09±0.01 | ND | 1954.12±41.47 |
|  | ZD49 | 0.04±0.00 | ND | 1454.12±27.85 |
| *Lactobacillus hilgardii* | ZD42 | 0.01±0.00 | ND | 1354.12±29.48 |

^a^ ND means not detected.


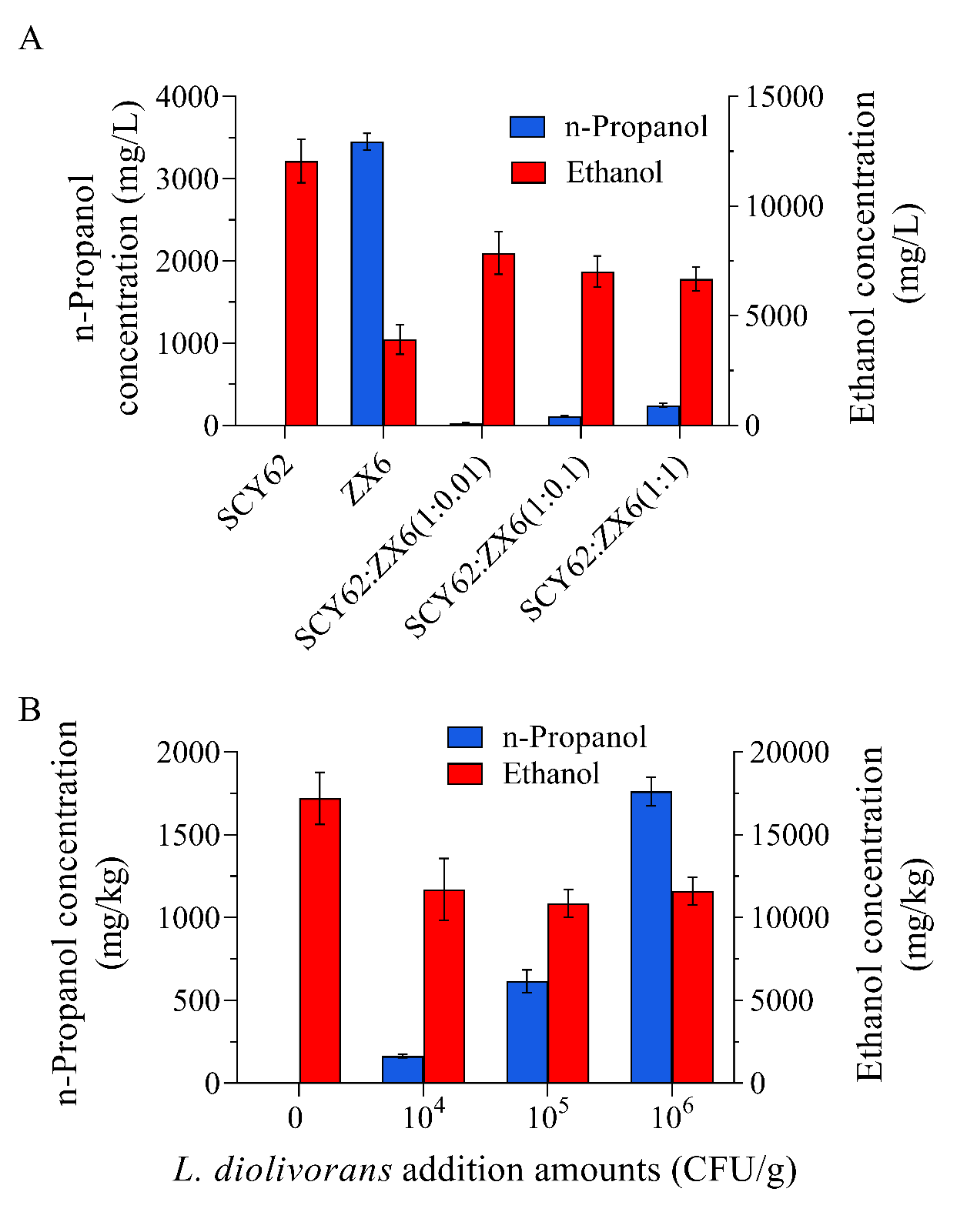
**Supplementary figures**

**Fig. S1.** n-Propanol and ethanol yields in co-culture fermentation of *L. diolivorans* ZX6 and *S. cerevisiae* SCY62. (A) Liquid co-culture. (B) Solid-state co-culture.

**Fig. S2.**
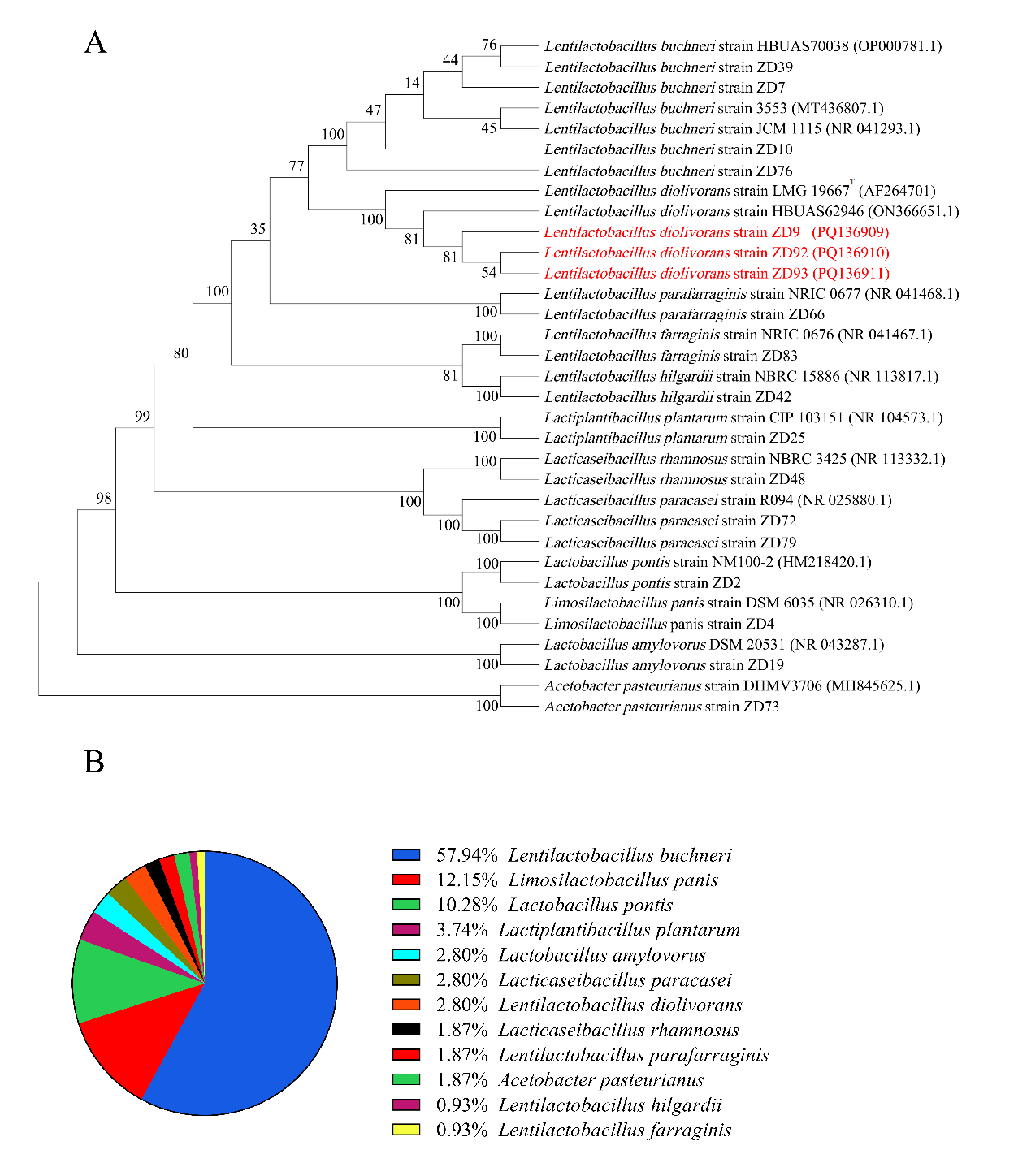
 Isolation and identification of the LAB and analysis of the proportion in SMRS medium from the SFBJ first round of fermented grains. (A) Phylogenetic tree of the LAB based on 16S rRNA gene sequences, (B) the proportion of each LAB species.


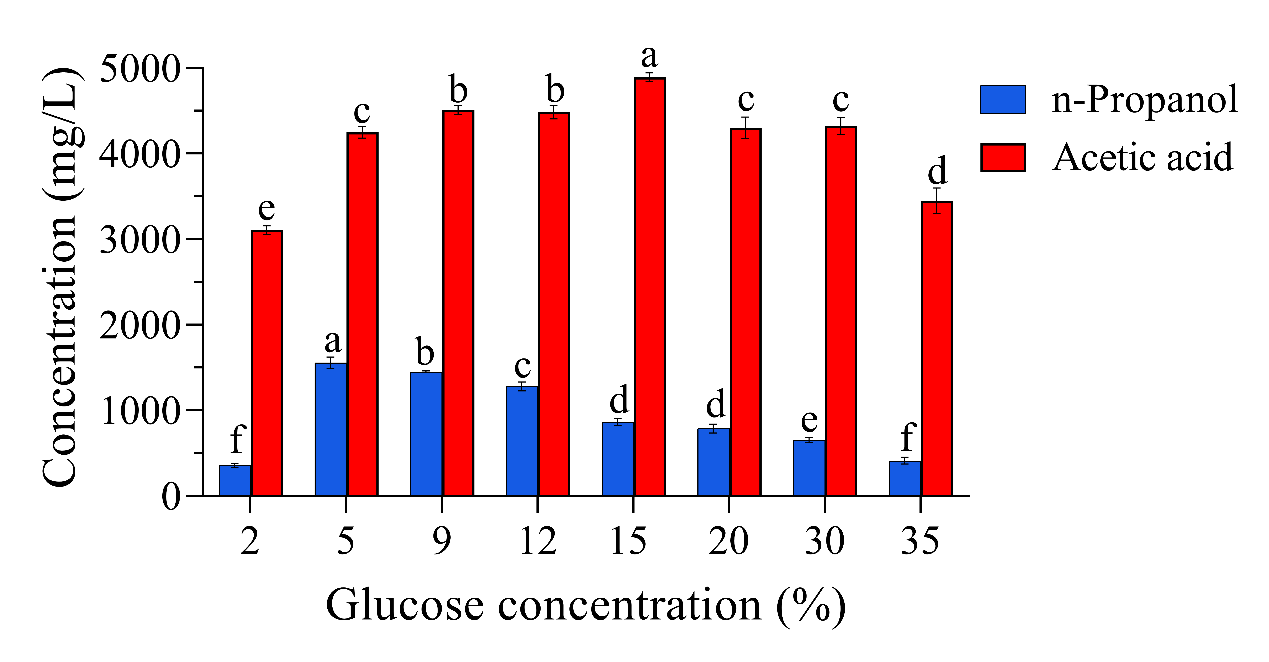
**Fig. S3.** The effects of glucose concentration on the yield of n-propanol fermented by *L. diolivorans* ZX6.

**

Fig. S4.** Significance analysis of the transcription levels of 42 genes associated with 24 enzymes in the metabolic pathway under different carbon source conditions (F: fructose, G: glucose, S: sucrose, X: xylose). *, 0.01 < P ≤ 0.05; **, 0.001 < P ≤ 0.01; ***, P ≤ 0.001.
